# Effect of Drying Process, Encapsulation, and Storage on the Survival Rates and Gastrointestinal Resistance of *L. salivarius* spp. *salivarius* Included into a Fruit Matrix

**DOI:** 10.3390/microorganisms8050654

**Published:** 2020-04-30

**Authors:** Ester Betoret, Noelia Betoret, Laura Calabuig-Jiménez, Cristina Barrera, Marco Dalla Rosa

**Affiliations:** 1 Instituto de Agroquímica y Tecnología de Alimentos, Consejo Superior de Investigaciones Científicas, 46980 Paterna, Spain; 2 Instituto de Ingeniería de Alimentos para el Desarrollo, Universitat Politècnica de València, 46022 Valencia, Spain; noebeval@tal.upv.es (N.B.); laucajim@upvnet.upv.es (L.C.-J.); mcbarpu@tal.upv.es (C.B.); 3 Department of Agriculture and Food Sciences, University of Bologna, 74521 Cesena, Italy; marco.dallarosa@unibo.it; 4 Interdepartmental Centre for Agri-Food Industrial Research, University of Bologna, 47521 Cesena, Italy

**Keywords:** microencapsulation, hot air drying, freeze drying, probiotic, gastrointestinal simulation

## Abstract

In a new probiotic food, besides adequate physicochemical properties, it is necessary to ensure a minimum probiotic content after processing, storage, and throughout gastrointestinal (GI) digestion. The aim of this work was to study the effect of hot air drying/freeze drying processes, encapsulation, and storage on the probiotic survival and in vitro digestion resistance of *Lactobacillus salivarius* spp. *salivarius* included into an apple matrix. The physicochemical properties of the food products developed were also evaluated. Although freeze drying processing provided samples with better texture and color, the probiotic content and its resistance to gastrointestinal digestion and storage were higher in hot air dried samples. Non-encapsulated microorganisms in hot air dried apples showed a 79.7% of survival rate versus 40% of the other samples after 28 days of storage. The resistance of encapsulated microorganisms to in vitro digestion was significantly higher (*p* ≤ 0.05) in hot air dried samples, showing survival rates of 50–89% at the last stage of digestion depending on storage time. In freeze dried samples, encapsulated microorganisms showed a survival rate of 16–47% at the end of digestion. The different characteristics of the food matrix after both processes had a significant effect on the probiotic survival after the GI digestion. Documented physiological and molecular mechanisms involved in the stress response of probiotic cells would explain these results.

## 1. Introduction

Juices, fruits, vegetables, breads, and cereals fiber snacks are the main options being studied in the area of probiotic functional foods as an alternative to dairy products [1]. It is generally accepted that to have an evident effect, probiotic foods should have a minimum concentration of 10^6^ CFU/mL or gram, or a total consumption of 10^8^–10^9^ colony forming units (CFU) per day depending on the microorganism strain and the physiological conditions of the host [2]. Therefore, that the probiotic microorganisms would exert a beneficial effect on the host, they should survive the stresses suffered during all food production processing and storage but they also should resist the passage through the gastrointestinal tract to be delivered in the active form to the specific target point [3].

In the development of probiotic functional foods, studies have shown that a specific food matrix can simultaneously allow the multiplication of probiotics and provide protection during the product shelf life [4,5]. Milk is considered the best matrix due to the high availability of all nutrients needed to probiotic microorganism growth. However, the fruit matrix has been demonstrated to be a suitable vehicle for the growth of probiotics [6]. The incorporation of probiotics into a fruit matrix, besides developing a new probiotic food products category, could protect the microorganisms from the stresses suffered during processing, storage, and the gastrointestinal (GI) digestion [6]. The encapsulation of probiotics could offer an additional protection of the microorganisms to maintain survival rates and higher viabilities during the GI digestion [7,8]. In addition, probiotic microorganisms are able to adapt to adverse conditions when they have been previously subjected to controlled stresses [9]. Vacuum impregnation technology can incorporate probiotic microorganisms into a fruit matrix in a controlled way without negative effects on the physicochemical and quality characteristics of the fresh fruits [10]. The encapsulation of microorganisms by high pressures homogenization allows obtaining homogeneous capsules, sphere shaped and quite rough, with diameters under 100 µm [11]. However, the high water content and water activity of the fresh fruits require following a stabilization process when they are not going to be consumed fresh and will be stored for long periods. Drying permits increasing the product shelf life by reducing the water activity, therefore, limiting the development of pathogenic microorganisms and conferring specific characteristics [12]. Freeze drying technology is the preference selection technique when related to probiotic microorganisms as low temperatures are used. The freeze dried food products maintain stable during long periods along with having no negative changes in color neither in volume of the samples [13]. However, freeze drying technology is expensive and the resulting products have high porosity levels, which could affect the survival of the microbial cells during the GI digestion. Otherwise, hot air drying technology is not expensive as freeze drying but the products can reach higher temperatures with significant changes in color and volume. Moreover, in hot air dried products the shrinkage of the samples, together with the velocity of water elimination can promote chemical reactions, changes, and interactions among compounds than can have an effect on their stability and degradation [14]. The aim of this work was to study the effect of the two drying processes most commonly applied in the food industry, encapsulation, and storage on the physicochemical properties, the probiotic survival, and in vitro digestion resistance of *Lactobacillus salivarius* spp. *salivarius* included into an apple matrix. 

## 2. Materials and Methods

### 2.1. Raw Materials

The microorganism *Lactobacillus salivarius* spp. *salivarius* was provided in a freeze dried form by the Spanish Type Cultures Collection (CECT 4063) (Valencia, Spain) [15]. 

Low pulp mandarin juice, from mandarin fruits cv. *Ortanique* (*Citrus sinensis* × *Citrus reticulata*) acquired from a local farmer cooperative located in Benaguacil (Valencia, Spain), was obtained following the methodology previously described in [14]. 

Apples (cv. *Granny Smith*) were acquired from a local market located in Valencia (Valencia, Spain). Apple discs that are 5 mm thick, 20 mm of internal, and 60 mm external diameter were used in this study. 

### 2.2. Microencapsulation Process

The *L. salivarius* spp. *salivarius* microorganisms were encapsulated by homogenization pressures using the methodology described by [16] with some modifications. Briefly, a mixture of 25 mL of microorganism solution containing 10^10^ CFU/mL of *L. salivarius* isolated by centrifugation at 7700× *g* for 15 min at 10 °C (Beckman Coulter AvantiTM J-25, Brea, CA, USA), 100 mL of sodium alginate (3%) (Sigma-Aldrich, Steinheim, Germany), 1 mL of tween 80 (Sharlau, Sentmenat, Spain), and 200 mL of commercial sunflower oil was homogenized at 70 MPa in two passes (Panda Plus Niro Soavi, Parma, Italy). Calcium chloride 0.1 M (Sigma-Aldrich, Steinheim, Germany) was used to break the emulsion and kept overnight at 4 °C to separate the phases. Microcapsules were isolated from the liquid phase by centrifugation at 7700× *g* for 15 min at 10 °C (Beckman Coulter AvantiTM J-25, Brea, CA, USA). 

### 2.3. Incorporation of Microorganisms into the Food Matrix and Drying Process

**Figure 1:**
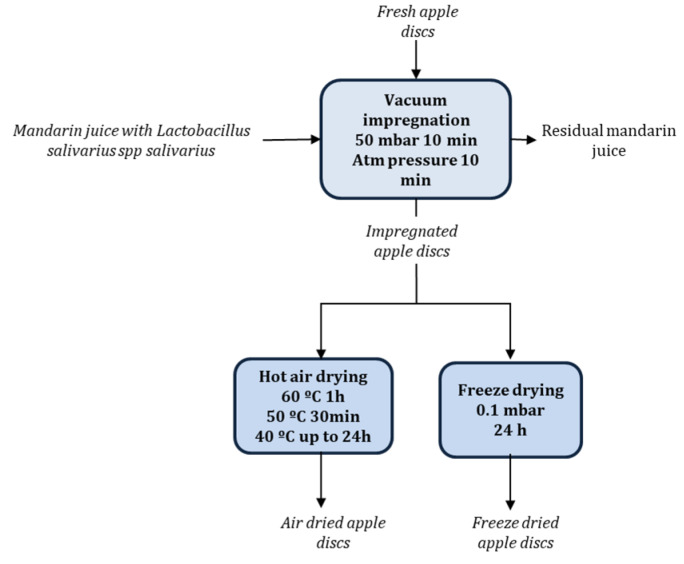
Schematic process to obtain impregnated and dried apple discs with probiotic microorganisms.

Mandarin juices were used as a probiotic liquid vehicle and apple discs were chosen as the food matrix. Mandarin juices containing 10^9^ CFU/mL of *L. salivarius* spp. *salivarius* encapsulated and non-encapsulated were prepared as described in [17]. Mandarin juices with probiotic microorganisms were incorporated into the structural matrix of apple by vacuum impregnation with a vacuum pressure of 50 mbar for 10 min and atmospheric pressure for further 10 min. The proportion of mandarin juice and apple was 4:1 (*w*/*w*), with apple discs totally submerged into the mandarin juice with probiotic microorganisms. Air dried apple samples with *L. salivarius* spp. *salivarius* encapsulated and non-encapsulated were obtained using an air drier (POL-EKO model CLW400 TOP, Controltecnica Instrumentación Científica S.L., Madrid, Spain). The air drying operation was carried out with multiple temperature stages in one experiment, 60 °C for 1 h, 50 °C for 30 min, and 40 °C up to 24 h. Freeze dried apple samples with *L. salivarius* spp. *salivarius* encapsulated and non-encapsulated were obtained by freezing at −40 °C for 24 h and freeze dried (Telstar, Lyoalfa-6, Azbil, Spain) at 0.1 mbar for 24 h. The results provided are the average of three replicates (Figure 1).

### 2.4. Determination of Physicochemical Properties

Dried apple discs were characterized in terms of pH, water activity, and moisture content. Water activity was measured with a dewpoint hygrometer (Aqualab 4TE; Decagon devices, Pullman, WA, USA). To determine the pH, an aqueous solution 1:1 (*w*/*w*) was prepared and then measured with a digital pH-meter (Mettler Toledo Gmbh., Schwerzenbach, Switzerland). Moisture content was calculated from the water lost by a known amount of sample at 60 °C for 24 h and further 60 °C at −0.8 bar (Vaciotem, P-SELECTA, Barcelona, Spain) until a constant weight. Determinations were performed in triplicate.

### 2.5. Texture and Color Measurements

Color was measured using a spectrocolorimeter (MINOLTA model CM-1000R), with an illuminant D65 and a 10° angle of vision observer. Results were provided in the CIE L*a*b* color system where L* defines lightness, a* denotes the red-green value, and b* the yellow-blue value. The hue (h*ab) and the chroma (C*ab) of the samples were also obtained. The results provided are the average of four replicates.

The mechanical properties of dried apples were evaluated in a texturometer (Stable Micro Systems mod. TA.XT plus Godalming, Surrey GU7 1YL, United Kingdom) by means of a compression test performed with a 2 mm diameter punch on the flat side of each apple disc. Maximum force (N) was the parameter used to characterize the samples. The equipment was configured so that the probe penetrated completely the sample by covering a distance of 10 mm. Data were collected with the software Textura exponent 32, version 6.1.2.0. The speed of the assay was 2.0 mm/s, with an activation force of 0.04903 N, and cell charge of 5 kg. The results provided are the average of six replicates.

### 2.6. Microorganisms Content Determination

*L. salivarius* spp. *salivarius* was determined on a double layer MRS agar (Scharlab, Barcelona, Spain) after incubation at 37 °C for 24 h. In the encapsulated samples, the first dilution was done in a phosphate buffer solution stirred for 30 min. Values provided are the average of four replicates. 

### 2.7. Gastrointestinal Simulation Process

In order to simulate the effect of the gastrointestinal digestion on the probiotic microorganisms, the methodology described in [17] was followed. T_i_ referred to a *L. salivarius* spp. *salivarius* content (CFU/g), meanwhile t_i_ referred to a moment in the gastrointestinal digestion. Ten grams of the sample were mixed with 10 mL of pepsine 0.6% (*w*/*v*) (Sigma-Aldrich, Steinheim, Germany) adjusted to pH 3 with HCl 4M (t_1_—T_1_) and stirred at 37 °C for 90 min (t_2_—T_2_). The addition on a phosphate buffer solution (pH 8) with 10% of bile (Sigma-Aldrich, Steinheim, Germany) was performed in the third step (t_3_—T_3_). The phosphate buffer solution with 0.3% of bile and 0.1% pancreatine (Sigma-Aldrich, Steinheim, Germany) was added on following an incubation at 37 °C for 90 min (t_4_—T_4_). The results provided are the average of four replicates.

### 2.8. Storage

Dried samples were stored in hermetically closed opaque plastic bags without head space at room temperature until analysis. The samples were analyzed weekly during the 28 days.

### 2.9. Statistical Analysis

The statistical analysis was carried out by a multifactorial ANOVA analysis, at 95% confidence level, using the Statgraphics centurion XVI software (StatPoint Technologies, Warrenton, VA, USA).

## 3. Results and Discussion

### 3.1. Physicochemical Properties

The physicochemical properties of the air dried and freeze dried apple discs containing *L. salivarius* spp. *salivarius* encapsulated and non-encapsulated are presented in Table 1. 

The pH, water activity, and moisture content values were similar to that obtained in previous studies [6]. All the variables studied, drying process, encapsulation, and storage time, had a significant effect (*p* ≤ 0.05) on the water activity values, which were higher for the air dried samples with encapsulated *L. salivarius* spp. *salivarius*. Moreover, the statistical analysis revealed an interaction effect between all the variables studied. The effect of the storage was more evident in the freeze dried samples with values going from 0.23 to 0.398 in samples with encapsulated *L. salivarius* spp. *salivarius* and from 0.381 to 0.433 in samples with non-encapsulated *L. salivarius* spp. *salivarius*. However, in air dried samples the water activity values remained stable going from 0.56 to 0.554 and from 0.454 to 0.453 in samples with encapsulated and non-encapsulated *L. salivarius* spp. *salivarius,* respectively. The low water activity values obtained in the samples dehydrated by both techniques did not allow the growth of harmful fungi or other microorganisms during the storage time. The moisture content was significantly affected (*p* ≤ 0.05) by the drying process and the storage time but not by the presence of *L. salivarius* spp. *salivarius* encapsulated or non-encapsulated. The statistical analysis revealed an interaction effect between the drying process-storage and encapsulation-storage variables. The higher values of moisture were obtained in air dried samples with *L. salivarius* spp. *salivarius* non-encapsulated. As for the water activity, the variation of values during the storage were higher in freeze dried samples. Freezing and sublimation of water during the freeze drying process causes greater cell disruption and breakage, resulting in higher porosity samples. This facilitates the loss of water during the drying process and increases the gain of water during storage. The pH values were significantly affected (*p* ≤ 0.05) by all the variables studied. The statistical analysis also revealed an interaction effect. In all cases the pH values ranged between 3.59 and 4.076. The small differences observed can be attributed to the small differences in the growth of bacteria or to the small differences in humidity. These differences are not relevant to the physicochemical properties and stability of the final product.

The values of color coordinates were significantly higher in freeze dried samples (Table 2). The L^*^ coordinate was significantly affected by the drying process but not by encapsulation nor storage time. The observed brightness values in air dried samples can be explained by taking into account the changes occurring in tissue structure during drying, mainly decreasing porosity, shrinkage, and destructuring [18,19]. A significant increase in the brightness value of the freeze dried samples was observed due to the lower water content and the porous structure formation. The coordinate a^*^ value was, in all cases, considerably lower than the coordinate b^*^ indicating that the characteristic yellow color of the fresh fruit was maintained. However, an increase in the coordinates a^*^ and b^*^ samples revealed a tendency to an orange color for the air dried samples with non-encapsulated microorganisms as a result of oxidation reactions mainly because of long exposure to air and browning enzymatic reactions because of the use of high temperatures. The lower increase in the a* coordinate by the freeze dried samples can be attributed to the use of low temperatures during the process and the absence of air exposure during the sublimation of frozen water. Statistical analysis of the data showed a significant effect of the drying process and encapsulation in coordinates a^*^, b^*^, C^*^_ab_. A significant effect of the storage time was also observed on the coordinate h^*^_ab_. The coordinate h^*^_ab_ showed a significant interaction effect between the drying process and storage. The samples that maintained a more stable form of the h^*^_ab_ coordinate during the storage were the encapsulated freeze dried samples.

The maximum force required to penetrate the samples was significantly affected by all the variables analyzed, showing an interaction effect between the drying—encapsulation and encapsulation—storage variables (Table 1). The main changes induced by dehydration treatments that affected the mechanical properties of plant tissues were cellular turgor loss, alteration of the middle lamella, altered cell wall strength, changes in the volume fractions of gas and liquid, as well as changes in the size and shape of the samples [20]. The values of maximum force obtained in air dried samples were significantly higher with remarkable values in non-encapsulated samples. This may be explained in terms of lower water content in the freeze dried samples that reached at the end of the process, but also in structural changes occurring in different plant tissues depending on the stabilization technique employed. Thus, the removal of frozen water by sublimation promoted the formation of a porous structure, while the associated water evaporation involving convective drying resulted in greater volume changes and promoted the collapse of its structure [21]. The presence of encapsulated microorganisms modified significantly (*p* ≤ 0.05) the texture of air dried samples, with lower values of maximum force similar to those of freeze dried samples. The incorporation of alginate capsules with a low density into the structure of apple discs probably led to a reduction in their hardness as previously notified in other food matrices [22]. In air dried samples the addition of microcapsules resulted in less dense crystalline networks and reduced particle–particle interactions, with more open structures and void spaces [23].

### 3.2. Content of L. salivarius spp. salivarius after Drying and during Storage

The content of *L. salivarius* spp. *salivarius* encapsulated and non-encapsulated in dried apples together with the survival percentage during the storage is shown in Table 3. The drying process, encapsulation of microorganisms, and storage had a significant effect (*p* ≤ 0.05) on the content and survival of *L. salivarius* spp. *salivarius*. Immediately after drying, the microbial content was higher in the freeze dried samples. Until 14 days of storage, the survival percentage in all samples was higher than 50%. The significant damage caused to cells during dehydration operations, together with the limited availability of water and nutrients, causes a significant decrease in the content of microorganisms within a few days of storage. The higher content of non-encapsulated *L. salivarius* spp. *salivarius* observed in air dried samples during all the storage time was remarkable. The content of *L. salivarius* spp. *salivarius* obtained at 14 and 28 days of storage were 5.66 and 5.14 Log CFU/g_dried_, respectively (Table 3). These values are significantly different from those obtained previously [6] in air dried samples at 40 °C for 24 h, being 2.89 and 1.3 Log CFU/g_dried_ after 14 and 30 days of storage. The differences obtained in both studies could be attributed to the different drying temperatures and could be explained by taking into account the responses of lactobacilli to specific stresses and their capacity of adaptation and survival. The probiotic microorganisms contained in our apple samples faced several stresses during both drying processes. Drying temperatures, acid pH, osmotic pressures, and oxidation physiologically affected the cells. The responses of lactobacilli to environmental stress have been reported [9]. The physiological and molecular mechanisms involved in the stress response are well documented, including the induction of a specific protein leading to possible increases in specific or multiple stress tolerances [24]. In our case, the high temperatures (60 and 50 °C) during air drying to which the microbial cells have been subjected for a short period of time together with the oxidation conditions could have favored their survival during the subsequent air drying and storage. Our results agree with [25] who found an increase in the *L. salivarius* spp. *salivarius* survival by heating bacterial cultures at 50 °C for 15 min before spray-drying. The heat stress response could have an effect on the technological performance of lactobacilli due to the induction of some metabolic pathways [26]. The induction of these responses could be found after exposure to sublethal heat stress (specific response) or after exposure to other types of environmental stress (generic response), as was previously proven in other probiotic microorganisms [26]. The porous envelope of the encapsulated microorganisms could confer a protection against high temperatures, avoiding the favorable response to thermal stress, but seemed to not protect the cells against the oxidation or the water migrations during the storage. This was in agreement with other authors that observed alginate capsules were not able to protect encapsulated microorganisms from water migrations [27] or oxidation reactions [28] during storage.

### 3.3. Content of L. salivarius spp. salivarius after the Gastrointestinal (GI) Digestion Process

**Figure 2:**
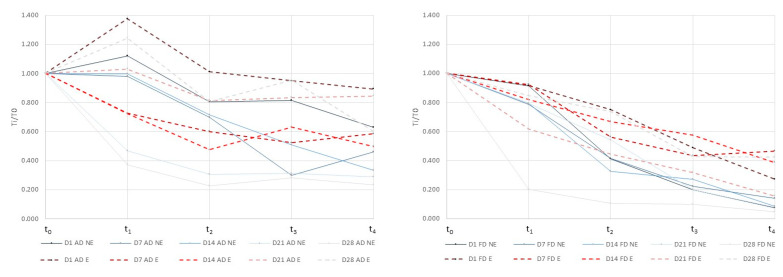
Relative content (T_i_/T_0_ = CFU in t_i_/CFU in t_0_) of *L. salivarius* spp. *salivarius* at each stage of the GI digestion process in air dried (left) and freeze dried (right) samples.

The simulated GI digestion process in this study covered the critical steps suffered by the probiotic microorganisms throughout the entire digestive system [17]. Thus, the t_1_ and t_2_ steps referred to the gastric mixture and digestion, respectively, t_3_ referred to the duodenal shock and t_4_ referred to the intestinal digestion. As a probiotic microorganism, to exert a beneficial effect, *L. salivarius* spp. *salivarius* should survive until the last step of the GI digestion (t_4_). *L. salivarius* spp. *salivarius* has been demonstrated to have a potential effect against the *Helicobacter pylori* infection [15]. In order to exert a specific beneficial effect against the *Helicobacter pylori* infection, the microorganisms should be able to colonize the stomach (t_2_). The content of *L. salivarius* spp. *salivarius* encapsulated and non-encapsulated in dried apples, exposed to simulated gastrointestinal tract conditions is presented in Table 4. All the variables studied, drying operation, encapsulation of the microorganisms, storage, GI digestion process, as well as the interaction between all the variables indicated, had a significant effect (*p* ≤ 0.05) on the microorganism content. In order to easily observe the differences obtained among samples, the relative content (T_i_/T_0_ = CFU in t_i_/CFU in t_0_) of *L. salivarius* spp. *salivarius* at each stage of the GI digestion process in air dried (left) and freeze dried (right) samples is presented in Figure 2. The microbial content after simulation of the GI tract conditions was higher in air dried samples than in freeze dried ones. The authors compared the survival of different probiotic microorganisms to the GI digestion in cheese, orange juice, and bread, and found that the strain selected independently of the food matrix had an influence on the survival of microorganisms [29]. However, the composition with more fat content and the structure, liquid, or solid, of the different food matrices affected the probiotic survival [29]. As far as we know, there are no studies that compare the same food matrix subjected to different processes. In our case, the different characteristics of the food matrix after both processes had a significant effect on the probiotic survival after GI digestion. The encapsulation of *L. salivarius* spp. *salivarius* was effective in protecting microbial cells during digestion processing, and the protection was higher during the storage in both dried samples. Generally, encapsulation showed higher survival rates in the t_1_ and t_3_ stages independently of the drying process applied. However, in the first stage of the digestion process, the positive effect of the encapsulation on the freeze dried samples only appears after 14 days of storage. Our findings were similar to other studies in which the encapsulation increased the survival of the probiotics microorganisms during the GI digestion and the storage [30,31]. The survival rate reached by the air dried samples in the t_1_ stage was remarkable including a growth of microorganisms in some cases. The mandarin juice used as a liquid vehicle for microorganism growth and for their incorporation into the apple matrix had a pH of 3.4. Apple fruit had a pH between 3 and 4. Probably, the growing, processing, and storage of *L. salivarius* spp. *salivarius* in acidic conditions could promote the acid tolerance of microbial cells [32]. The porosity and volume differences between air and freeze dried samples could explain the differences obtained in the microorganisms survival rates at t_1_ in both drying processes. Less porosity in air dried samples together with marked shrinkages could promote a slower contact kinetics and could favor the survival of *L. salivarius* spp. *salivarius* in acidic conditions. This agrees with the results obtained by [33] who demonstrated that two lactobacillus strains were able to survive in artichokes 90 days without showing viability loss after the GI digestion and concluded that the roughness of the vegetable structure contributed to the successful bacteria protection. 

## 4. Conclusions

Hot air and freeze drying allowed obtaining of dried apples that contain probiotic microorganisms with suitable physicochemical characteristics during 28 days. Contrary to that generally expected, the hot air drying processing allowed obtaining a higher amount of probiotic microorganisms in fruit matrices than freeze drying and also with a higher resistance to the GI digestion. The different characteristics of the food matrix after both processes had a significant effect on the probiotic survival after the GI digestion. An appropriate managing of the process operations could help increase the microorganism survival until acceptable levels of probiotics microorganism. The understanding of the processes effect on the microorganisms’ survival and its resistance to in vitro digestion are key steps to develop this new type of product. Further fundamental studies on probiotic survival during processing and storage would be necessary in order to enhance the microorganisms’ protection and thus, the industrial utility.

## Figures and Tables

**Table 1:** Physicochemical characteristics (pH, water activity (a_w_), and moisture content (x_w_)) and F_max_ of dried apples with encapsulated and non-encapsulated microorganisms during 28 days of storage. Results expressed as mean ± standard deviation of at least three replicates.

		Storage (days)	a_w_	x_w_(kg_water_/kg_sample_)	pH	F_max_
**Air Drying**	Encapsulated	1	0.56 ± 0.02	0.111 ± 0.009	3.67 ± 0.03	7 ± 2
		7	0.553 ± 0.003	0.103 ± 0.012	3.66 ± 0.02	6.1 ± 0.6
		14	0.527 ± 0.003	0.130 ± 0.019	3.62 ± 0.01	7.9 ± 0.7
		21	0.541 ± 0.003	0.114 ± 0.001	3.78 ± 0.01	7.1 ± 1.6
		28	0.554 ± 0.004	0.101 ± 0.006	3.64 ± 0.02	7.9 ± 0.7
	Non-Encapsulated	1	0.454 ± 0.015	0.142 ± 0.016	3.65 ± 0.02	14.4 ± 1.5
		7	0.463 ± 0.01	0.114 ± 0.050	3.67 ± 0.02	14.8 ± 1.8
		14	0.455 ± 0.004	0.099 ± 0.006	3.75 ± 0.012	13.3 ± 1.6
		21	0.480 ± 0.018	0.105 ± 0.004	3.78 ± 0.010	17 ± 4
		28	0.453 ± 0.009	0.094 ± 0.008	3.67 ± 0.02	17 ± 4
**Freeze Drying**	Encapsulated	1	0.23 ± 0.02	0.059 ± 0.008	4.076 ± 0.016	5.8 ± 0.9
		7	0.37 ± 0.02	0.044± 0.007	3.59 ± 0.02	3.8 ± 1.6
		14	0.367 ± 0.003	0.085 ± 0.005	3.52 ± 0.02	2.7 ± 0.5
		21	0.385 ± 0.014	0.053 ± 0.002	3.58 ± 0.02	5.3 ± 1.7
		28	0.398 ± 0.007	0.084 ± 0.005	3.61 ± 0.05	6.4 ± 1.8
	Non-Encapsulated	1	0.381 ± 0.012	0.05 ± 0.02	3.88 ± 0.04	2.4 ± 0.3
		7	0.415 ± 0.003	0.032 ± 0.002	3.780 ± 0.012	4.3 ± 0.5
		14	0.408 ± 0.004	0.049 ± 0.003	3.845 ± 0.006	4.3 ± 1.2
		21	0.42 ± 0.01	0.068 ± 0.012	4.01 ± 0.02	6.79 ± 1.14
		28	0.433 ± 0.009	0.049 ± 0.002	3.940 ± 0.012	5 ± 2
***p*-Values**	Drying (D)		0.0000	0.0000	0.0000	0.0000
	Encapsulation (E)		0.0013	0.0589	0.0000	0.0000
	Storage (S)		0.0000	0.0328	0.0000	0.0001
	D*E		0.0000	0.0927	0.0000	0.0000
	D*S		0.0000	0.0150	0.0000	0.9067
	E*S		0.0000	0.0030	0.0000	0.0122

**Table 2:** Color coordinates (L*a*b*), chroma (C^*^_ab_), and hue (h^*^_ab_) of dried apples with encapsulated and non-encapsulated microorganisms during 28 days of storage. Results expressed as mean ± standard deviation of at least three replicates.

		Storage (Days)	L*	a*	b*	C^*^_ab_	h^*^_ab_
**Air Drying**	Encapsulated	1	43 ± 12	3 ± 2	19 ± 9	19 ± 9	81 ± 7
		7	48 ± 10	3.6 ± 0.4	24 ± 8	24 ± 8	81 ± 4
		14	61 ± 5	3.10 ± 1.09	29 ± 3	30 ± 3	84 ± 2
		21	40 ± 5	4 ± 2	20 ± 7	20 ± 7	77 ± 5
		28	53 ± 10	6 ± 2	29 ± 9	30 ± 10	78 ± 3
	Non-Encapsulated	1	55 ± 2	5.4 ± 1.2	31 ± 3	31 ± 3	80 ± 3
		7	62 ± 10	8 ± 3	33 ± 6	34 ± 7	76 ± 4
		14	47 ± 7	7 ± 3	26 ± 3	27 ± 3	75 ± 6
		21	47 ± 5	8.1 ± 1.6	26 ± 5	27 ± 4	72 ± 6
		28	40 ± 4	10.4 ± 1.6	22 ± 2	24 ± 2	64 ± 5
**Freeze Drying**	Encapsulated	1	82.9 ± 1.4	−0.4 ± 0.9	27.6 ± 0.9	27.6 ± 0.9	91 ± 2
		7	82.1 ± 0.6	−2.1 ± 0.3	27 ± 3	27 ± 3	94.60 ± 1.02
		14	81.1 ± 1.5	−1.1 ± 1.2	25 ± 4	25 ± 4	93 ± 3
		21	81.3 ± 0.9	−2.0 ± 0.6	27 ± 5	28 ± 5	94.3 ± 1.5
		28	80.7 ± 1.6	0.1 ± 1.3	31 ± 4	31 ± 4	90 ± 3
	Non-Encapsulated	1	78.5 ± 0.9	3.1 ± 0.5	37 ± 4	37 ± 4	85.1 ± 0.3
		7	78 ± 2	5 ± 2	37 ± 3	37 ± 3	83 ± 3
		14	78 ± 3	4.2 ± 1.3	37 ± 2	37 ± 2	83 ± 2
		21	78.1 ± 1.8	4.4 ± 1.9	38 ± 4	39 ± 4	84 ± 3
		28	78.3 ± 1.4	5 ± 2	38 ± 3	38 ± 3	82 ± 3
***p*-Values**	Drying (D)		0.0000	0.0000	0.0000	0.0000	0.0000
	Encapsulation (E)		0.4986	0.0000	0.0000	0.0000	0.0000
	Storage (S)		0.0721	0.0004	0.6644	0.5766	0.0005
	D*E		0.0933	0.0351	0.0073	0.0150	0.2208
	D*S		0.0790	0.0951	0.2234	0.2813	0.0197
	E*S		0.0060	0.2359	0.0181	0.0308	0.0683

**Table 3:** Microbial content of *L. salivarius* spp. *salivarius* encapsulated and non-encapsulated (Log CFU/g_dried_) in dried apples stored for 28 days. The number in brackets indicates the survival of microorganisms in percentage. Results expressed as mean ± standard deviation of four replicates.

		Day 1	Day 7	Day 14	Day 21	Day 28
**Air Drying**	Encapsulated	6.28 ± 0.09 (100)	5.030 ± 0.106 (80)	3.9 ± 0.3 (62)	3.36 ± 0.12 (53.5)	2.54 ± 0.06 (40.4)
	Non-Encapsulated	6.45 ± 0.09 (100)	6.19 ± 0.04 (96)	5.66 ± 0.06 (87.8)	5.36 ± 0.04 (83)	5.14 ± 0.08 (79.7)
**Freeze Drying**	Encapsulated	7.00 ± 0.13 (100)	5.01 ± 0.04 (71.6)	3.61 ± 0.07 (51.6)	3.86 ± 0.06 (55)	2.64 ± 0.13 (37.7)
	Non-Encapsulated	7.10 ± 0.04 (100)	5.40 ± 0.07 (76)	5.13 ± 0.05 (72.3)	3.80 ± 0.05 (53.5)	3.2 ± 0.2 (45.1)

The obtained *p*-values of variables (drying, encapsulation, and storage) and their interactions were in all cases 0.0000.

**Table 4:** Microbial content of *L. salivarius* spp. *salivarius* encapsulated and non-encapsulated (Log CFU/g_dried_) during the digestion process of dried apples stored for 28 days of storage. The number in brackets indicates the survival of microorganisms in percentage. Results expressed as mean ± standard deviation of four replicates.

			Day 1	Day 7	Day14	Day 21	Day 28
**Air Drying**	**Encapsulated**	t_0_	6.28 ± 0.09 (100)	5.030 ± 0.106 (100)	3.9 ± 0.3 (100)	3.36 ± 0.1 (100)	2.54 ± 0.06 (100)
		t_1_	8.6 ± 0.2 (138)	3.649 ± 0.103 (73)	2.84 ± 0.07 (72)	3.46 ± 0.06 (103)	3.16 ± 0.04 (124)
		t_2_	6.3 ± 0.2 (101)	3.02 ± 0.07 (60)	1.9 ± 0.2 (48)	2.73 ± 0.03 (81)	2.052 ± 0.104 (81)
		t_3_	6.0 ± 0.2 (95)	2.64 ± 0.06 (52)	2.48 ± 0.12 (63)	2.80 ± 0.04 (83)	2.42 ± 0.12 (95)
		t_4_	5.6 ± 0.3 (89)	2.94 ± 0.04 (59)	1.9 ± 0.3 (50)	2.84 ± 0.09 (84)	1.5 ± 0.2 (60)
	**Non-Encapsulated**	t_0_	6.45 ± 0.09 (100)	6.19 ± 0.04 (100)	5.66 ± 0.06 (100)	5.36 ± 0.04 (100)	5.14 ± 0.08 (100)
		t_1_	7.23 ± 0.04 (112)	6.06 ± 0.13 (98)	5.63 ± 0.09 (100)	2.50 ± 0.09 (47)	1.9 ± 0.2 (37)
		t_2_	5.18 ± 0.09 (80)	4.32 ± 0.05 (70)	4.04 ± 0.09 (71)	1.65 ± 0.09 (31)	1.2 ± 0.8 (23)
		t_3_	5.25 ± 0.05 (81)	1.85 ± 0.09 (30)	2.88 ± 0.06 (51)	1.673 ± 0.102 (31)	1.5 ± 0.9 (28)
		t_4_	4.046 ± 0.112 (63)	2.85 ± 0.04 (46)	1.9 ± 0.2 (34)	1.56 ± 0.04 (29)	1.2 ± 0.2 (23)
**Freeze Drying**	**Encapsulated**	t_0_	7.00 ± 0.13 (100)	5.01 ± 0.04 (100)	3.61 ± 0.07 (100)	3.86 ± 0.06 (100)	2.64 ± 0.13 (100)
		t_1_	6.40 ± 0.07 (91)	4.62 ± 0.13 (92)	2.95 ± 0.04 (82)	2.4 ± 0.2 (62)	2.2 ± 0.2 (85)
		t_2_	5.25 ± 0.09 (75)	2.8 ± 0.3 (56)	2.4 ± 0.5 (67)	1.7 ± 0.3 (44)	1.94 ± 0.13 (74)
		t_3_	3.42 ± 0.12 (49)	2.2 ± 0.2 (43)	2.1 ± 0.4 (58)	1.2 ± 0.3 (32)	1.13 ± 0.06 (43)
		t_4_	1.900 ± 0.102 (27)	2.3 ± 0.2 (47)	1.4 ± 0.2 (39)	0.6 ± 0.4 (16)	1.1 ± 0.2 (43)
	**Non-Encapsulated**	t_0_	7.10 ± 0.04 (100)	5.40 ± 0.07 (100)	5.13 ± 0.05 (100)	3.80 ± 0.05 (100)	3.2 ± 0.2 (100)
		t_1_	6.481 ± 0.112 (91)	4.242 ± 0.012 (79)	4.06 ± 0.12 (79)	3.02 ± 0.07 (79)	0.7 ± 0.7 (20)
		t_2_	2.93 ± 0.12 (41)	2.3 ± 0.2 (42)	1.7 ± 0.3 (33)	2.06 ± 0.14 (54)	0.3 ± 0.7 (10)
		t_3_	1.4 ± 0.9 (20)	1.2 ± 0.2 (22)	1.39 ± 0.05 (27)	0.8 ± 0.5 (20)	0.3 ± 0.7 (10)
		t_4_	0.5 ± 0.4 (8)	0.7 ± 0.2 (14)	0.4 ± 0.4 (9)	0.3 ± 0.3 (8)	0.2 ± 0.3 (5)

The obtained *p*-values of variables (drying, encapsulation, and storage) and their interactions were in all cases 0.0000.
